# Integrating students’ perspectives about online learning: a hierarchy of factors

**DOI:** 10.1186/s41239-020-00229-8

**Published:** 2020-12-02

**Authors:** Montgomery Van Wart, Anna Ni, Pamela Medina, Jesus Canelon, Melika Kordrostami, Jing Zhang, Yu Liu

**Affiliations:** Development for the JHB College of Business and Public Administration, 5500 University Parkway, San Bernardino, California 92407 USA

**Keywords:** Online education, Online teaching, Student perceptions, Online quality, Teaching presence, Cognitive presence, Student presence

## Abstract

This article reports on a large-scale (*n* = 987), exploratory factor analysis study incorporating various concepts identified in the literature as critical success factors for online learning from the students’ perspective, and then determines their hierarchical significance. Seven factors--Basic Online Modality, Instructional Support, Teaching Presence, Cognitive Presence, Online Social Comfort, Online Interactive Modality, and Social Presence--were identified as significant and reliable. Regression analysis indicates the minimal factors for enrollment in future classes—when students consider convenience and scheduling—were Basic Online Modality, Cognitive Presence, and Online Social Comfort. Students who accepted or embraced online courses on their own merits wanted a minimum of Basic Online Modality, Teaching Presence, Cognitive Presence, Online Social Comfort, and Social Presence. Students, who preferred face-to-face classes and demanded a comparable experience, valued Online Interactive Modality and Instructional Support more highly. Recommendations for online course design, policy, and future research are provided.

## Introduction

While there are different perspectives of the learning process such as learning achievement and faculty perspectives, students’ perspectives are especially critical since they are ultimately the raison d’être of the educational endeavor (Chickering & Gamson, 1987). More pragmatically, students’ perspectives provide invaluable, first-hand insights into their experiences and expectations (Dawson et al., 2019). The student perspective is especially important when new teaching approaches are used and when new technologies are being introduced (Arthur, 2009; Crews & Butterfield, 2014; Van Wart, Ni, Ready, Shayo, & Court, 2020). With the renewed interest in “active” education in general (Arruabarrena, Sánchez, Blanco, et al., 2019; Kay, MacDonald, & DiGiuseppe, 2019; Nouri, 2016; Vlachopoulos & Makri, 2017) and the flipped classroom approach in particular (Flores, del-Arco, & Silva, 2016; Gong, Yang, & Cai, 2020; Lundin, et al., 2018; Maycock, 2019; McGivney-Burelle, 2013; O’Flaherty & Phillips, 2015; Tucker*,* 2012) along with extraordinary shifts in the technology, the student perspective on online education is profoundly important. What shapes students’ perceptions of quality integrate are their own sense of learning achievement, satisfaction with the support they receive, technical proficiency of the process, intellectual and emotional stimulation, comfort with the process, and sense of learning community. The factors that students perceive as quality online teaching, however, has not been as clear as it might be for at least two reasons.

First, it is important to note that the overall online learning experience for students is also composed of non-teaching factors which we briefly mention. Three such factors are (1) convenience, (2) learner characteristics and readiness, and (3) antecedent conditions that may foster teaching quality but are not directly responsible for it. (1) Convenience is an enormous non-quality factor for students (Artino, 2010) which has driven up online demand around the world (Fidalgo, Thormann, Kulyk, et al., 2020; Inside Higher Education and Gallup, 2019; Legon & Garrett, 2019; Ortagus, 2017). This is important since satisfaction with online classes is frequently somewhat lower than face-to-face classes (Macon, 2011). However, the literature generally supports the relative equivalence of face-to-face and online modes regarding learning achievement criteria (Bernard et al., 2004; Nguyen, 2015; Ni, 2013; Sitzmann, Kraiger, Stewart, & Wisher, 2006; see Xu & Jaggars, 2014 for an alternate perspective). These contrasts are exemplified in a recent study of business students, in which online students using a flipped classroom approach outperformed their face-to-face peers, but ironically rated instructor performance lower (Harjoto, 2017). (2) Learner characteristics also affect the experience related to self-regulation in an active learning model, comfort with technology, and age, among others,which affect both receptiveness and readiness of online instruction. (Alqurashi, 2016; Cohen & Baruth, 2017; Kintu, Zhu, & Kagambe, 2017; Kuo, Walker, Schroder, & Belland, 2013; Ventura & Moscoloni, 2015) (3) Finally, numerous antecedent factors may lead to improved instruction, but are not themselves directly perceived by students such as instructor training (Brinkley-Etzkorn, 2018), and the sources of faculty motivation (e.g., incentives, recognition, social influence, and voluntariness) (Wingo, Ivankova, & Moss, 2017). Important as these factors are, mixing them with the perceptions of quality tends to obfuscate the quality factors directly perceived by students.

Second, while student perceptions of quality are used in innumerable studies, our overall understanding still needs to integrate them more holistically. Many studies use student perceptions of quality and overall effectiveness of individual tools and strategies in online contexts such as mobile devices (Drew & Mann, 2018), small groups (Choi, Land, & Turgeon, 2005), journals (Nair, Tay, & Koh, 2013), simulations (Vlachopoulos & Makri, 2017), video (Lange & Costley, 2020), etc. Such studies, however, cannot provide the overall context and comparative importance. Some studies have examined the overall learning experience of students with exploratory lists, but have mixed non-quality factors with quality of teaching factors making it difficult to discern the instructor’s versus contextual roles in quality (e.g., Asoodar, Vaezi, & Izanloo, 2016; Bollinger & Martindale, 2004; Farrell & Brunton, 2020; Hong, 2002; Song, Singleton, Hill, & Koh, 2004; Sun, Tsai, Finger, Chen, & Yeh, 2008). The application of technology adoption studies also fall into this category by essentially aggregating all teaching quality in the single category of performance *(*Al-Gahtani, 2016; Artino, 2010). Some studies have used high-level teaching-oriented models, primarily the Community of Inquiry model (le Roux & Nagel, 2018), but empirical support has been mixed (Arbaugh et al., 2008); and its elegance (i.e., relying on only three factors) has not provided much insight to practitioners (Anderson, 2016; Cleveland-Innes & Campbell, 2012).

## Research questions

Integration of studies and concepts explored continues to be fragmented and confusing despite the fact that the number of empirical studies related to student perceptions of quality factors has increased. It is important to have an empirical view of what students’ value in a single comprehensive study and, also, to know if there is a hierarchy of factors, ranging from students who are least to most critical of the online learning experience. This research study has two research questions.

The first research question is: *What are the significant factors in creating a high-quality online learning experience from students’ perspectives?* That is important to know because it should have a significant effect on the instructor’s design of online classes. The goal of this research question is identify a more articulated and empirically-supported set of factors capturing the full range of student expectations.

The second research question is: *Is there a priority or hierarchy of factors related to students’ perceptions of online teaching quality that relate to their decisions to enroll in online classes?* For example, is it possible to distinguish which factors are critical for enrollment decisions when students are primarily motivated by convenience and scheduling flexibility (minimum threshold)? Do these factors differ from students with a genuine acceptance of the general quality of online courses (a moderate threshold)? What are the factors that are important for the students who are the most critical of online course delivery (highest threshold)?

This article next reviews the literature on online education quality, focusing on the student perspective and reviews eight factors derived from it. The research methods section discusses the study structure and methods. Demographic data related to the sample are next, followed by the results, discussion, and conclusion.

## Literature review

Online education is much discussed (Prinsloo, 2016; Van Wart et al., 2019; Zawacki-Richter & Naidu, 2016), but its perception is substantially influenced by where you stand and what you value (Otter et al., 2013; Tanner, Noser, & Totaro, 2009). Accrediting bodies care about meeting technical standards, proof of effectiveness, and consistency (Grandzol & Grandzol, 2006). Institutions care about reputation, rigor, student satisfaction, and institutional efficiency (Jung, 2011). Faculty care about subject coverage, student participation, faculty satisfaction, and faculty workload (Horvitz, Beach, Anderson, & Xia, 2015; Mansbach & Austin, 2018). For their part, students care about learning achievement (Marks, Sibley, & Arbaugh, 2005; O’Neill & Sai, 2014; Shen, Cho, Tsai, & Marra, 2013), but also view online education as a function of their enjoyment of classes, instructor capability and responsiveness, and comfort in the learning environment (e.g., Asoodar et al., 2016; Sebastianelli, Swift, & Tamimi, 2015). It is this last perspective, of students, upon which we focus.

It is important to note students do not sign up for online classes solely based on perceived quality. Perceptions of quality derive from notions of the capacity of online learning when ideal—relative to both learning achievement and satisfaction/enjoyment, and perceptions about the likelihood and experience of classes living up to expectations. Students also sign up because of convenience and flexibility, and personal notions of suitability about learning. Convenience and flexibility are enormous drivers of online registration (Lee, Stringer, & Du, 2017; Mann & Henneberry, 2012). Even when students say they prefer face-to-face classes to online, many enroll in online classes and re-enroll in the future if the experience meets minimum expectations. This study examines the threshold expectations of students when they are considering taking online classes.

When discussing students’ perceptions of quality, there is little clarity about the actual range of concepts because no integrated empirical studies exist comparing major factors found throughout the literature. Rather, there are practitioner-generated lists of micro-competencies such as the Quality Matters consortium for higher education (Quality Matters, 2018), or broad frameworks encompassing many aspects of quality beyond teaching (Open and Distant Learning Quality Council, 2012). While checklists are useful for practitioners and accreditation processes, they do not provide robust, theoretical bases for scholarly development. Overarching frameworks are heuristically useful, but not for pragmatic purposes or theory building arenas. The most prominent theoretical framework used in online literature is the Community of Inquiry (CoI) model (Arbaugh et al., 2008; Garrison, Anderson, & Archer, 2003), which divides instruction into teaching, cognitive, and social presence. Like deductive theories, however, the supportive evidence is mixed (Rourke & Kanuka, 2009), especially regarding the importance of social presence (Annand, 2011; Armellini and De Stefani, 2016). Conceptually, the problem is not so much with the narrow articulation of cognitive or social presence; cognitive presence is how the instructor provides opportunities for students to interact with material in robust, thought-provoking ways, and social presence refers to building a community of learning that incorporates student-to-student interactions. However, teaching presence includes everything else the instructor does—structuring the course, providing lectures, explaining assignments, creating rehearsal opportunities, supplying tests, grading, answering questions, and so on. These challenges become even more prominent in the online context. While the lecture as a single medium is paramount in face-to-face classes, it fades as the primary vehicle in online classes with increased use of detailed syllabi, electronic announcements, recorded and synchronous lectures, 24/7 communications related to student questions, etc. Amassing the pedagogical and technological elements related to teaching under a single concept provides little insight.

In addition to the CoI model, numerous concepts are suggested in single-factor empirical studies when focusing on quality from a student’s perspective, with overlapping conceptualizations and nonstandardized naming conventions. Seven distinct factors are derived here from the literature of student perceptions of online quality: Instructional Support, Teaching Presence, Basic Online Modality, Social Presence, Online Social Comfort, cognitive Presence, and Interactive Online Modality.

### Instructional support

Instructional Support refers to students’ perceptions of techniques by the instructor used for input, rehearsal, feedback, and evaluation. Specifically, this entails providing detailed instructions, designed use of multimedia, and the balance between repetitive class features for ease of use, and techniques to prevent boredom. Instructional Support is often included as an element of Teaching Presence, but is also labeled “structure” (Lee & Rha, 2009; So & Brush, 2008) and instructor facilitation (Eom, Wen, & Ashill, 2006). A prime example of the difference between face-to-face and online education is the extensive use of the “flipped classroom” (Maycock, 2019; Wang, Huang, & Schunn, 2019) in which students move to rehearsal activities faster and more frequently than traditional classrooms, with less instructor lecture (Jung, 2011; Martin, Wang, & Sadaf, 2018). It has been consistently supported as an element of student perceptions of quality (Espasa & Meneses, 2010).

### Teaching presence

Teaching Presence refers to students’ perceptions about the quality of communication in lectures, directions, and individual feedback including encouragement (Jaggars & Xu, 2016; Marks et al., 2005). Specifically, instructor communication is clear, focused, and encouraging, and instructor feedback is customized and timely. If Instructional Support is what an instructor does before the course begins and in carrying out those plans, then Teaching Presence is what the instructor does while the class is conducted and in response to specific circumstances. For example, a course could be well designed but poorly delivered because the instructor is distracted; or a course could be poorly designed but an instructor might make up for the deficit by spending time and energy in elaborate communications and ad hoc teaching techniques. It is especially important in student satisfaction (Sebastianelli et al., 2015; Young, 2006) and also referred to as instructor presence (Asoodar et al., 2016), learner-instructor interaction (Marks et al., 2005), and staff support (Jung, 2011). As with Instructional Support, it has been consistently supported as an element of student perceptions of quality.

### Basic online modality

Basic Online Modality refers to the competent use of basic online class tools—online grading, navigation methods, online grade book, and the announcements function. It is frequently clumped with instructional quality (Artino, 2010), service quality (Mohammadi, 2015), instructor expertise in e-teaching (Paechter, Maier, & Macher, 2010), and similar terms. As a narrowly defined concept, it is sometimes called technology (Asoodar et al., 2016; Bollinger & Martindale, 2004; Sun et al., 2008). The only empirical study that did not find Basic Online Modality significant, as technology, was Sun et al. (2008). Because Basic Online Modality is addressed with basic instructor training, some studies assert the importance of training (e.g., Asoodar et al., 2016).

### Social presence

Social Presence refers to students’ perceptions of the quality of student-to-student interaction. Social Presence focuses on the quality of shared learning and collaboration among students, such as in threaded discussion responses (Garrison et al., 2003; Kehrwald, 2008). Much emphasized but challenged in the CoI literature (Rourke & Kanuka, 2009), it has mixed support in the online literature. While some studies found Social Presence or related concepts to be significant (e.g., Asoodar et al., 2016; Bollinger & Martindale, 2004; Eom et al., 2006; Richardson, Maeda, Lv, & Caskurlu, 2017), others found Social Presence insignificant (Joo, Lim, & Kim, 2011; So & Brush, 2008; Sun et al., 2008).

### Online social comfort

Online Social Comfort refers to the instructor’s ability to provide an environment in which anxiety is low, and students feel comfortable interacting even when expressing opposing viewpoints. While numerous studies have examined anxiety (e.g., Liaw & Huang, 2013; Otter et al., 2013; Sun et al., 2008), only one found anxiety insignificant (Asoodar et al., 2016); many others have not examined the concept.

### Cognitive presence

Cognitive Presence refers to the engagement of students such that they perceive they are stimulated by the material and instructor to reflect deeply and critically, and seek to understand different perspectives (Garrison et al., 2003). The instructor provides instructional materials and facilitates an environment that piques interest, is reflective, and enhances inclusiveness of perspectives (Durabi, Arrastia, Nelson, Cornille, & Liang, 2011). Cognitive Presence includes enhancing the applicability of material for student’s potential or current careers. Cognitive Presence is supported as significant in many online studies (e.g., Artino, 2010; Asoodar et al., 2016; Joo et al., 2011; Marks et al., 2005; Sebastianelli et al., 2015; Sun et al., 2008). Further, while many instructors perceive that cognitive presence is diminished in online settings, neuroscientific studies indicate this need not be the case (Takamine, 2017). While numerous studies failed to examine Cognitive Presence, this review found no studies that lessened its significance for students.

### Interactive online modality

Interactive Online Modality refers to the “high-end” usage of online functionality. That is, the instructor uses interactive online class tools—video lectures, videoconferencing, and small group discussions—well. It is often included in concepts such as instructional quality (Artino, 2010; Asoodar et al., 2016; Mohammadi, 2015; Otter et al., 2013; Paechter et al., 2010) or engagement (Clayton, Blumberg, & Anthony, 2018). While individual methods have been investigated (e.g. Durabi et al., 2011), high-end engagement methods have not.

Other independent variables affecting perceptions of quality include age, undergraduate versus graduate status, gender, ethnicity/race, discipline, educational motivation of students, and previous online experience. While age has been found to be small or insignificant, more notable effects have been reported at the level-of-study, with graduate students reporting higher “success” (Macon, 2011), and community college students having greater difficulty with online classes (Legon & Garrett, 2019; Xu & Jaggars, 2014). Ethnicity and race have also been small or insignificant. Some situational variations and student preferences can be captured by paying attention to disciplinary differences (Arbaugh, 2005; Macon, 2011). Motivation levels of students have been reported to be significant in completion and achievement, with better students doing as well across face-to-face and online modes, and weaker students having greater completion and achievement challenges (Clayton et al., 2018; Lu & Lemonde, 2013).

## Research methods

### Overview

To examine the various quality factors, we apply a critical success factor methodology, initially introduced to schools of business research in the 1970s. In 1981, Rockhart and Bullen codified an approach embodying principles of critical success factors (CSFs) as a way to identify the information needs of executives, detailing steps for the collection and analyzation of data to create a set of organizational CSFs (Rockhart & Bullen, 1981). CSFs describe the underlying or guiding principles which must be incorporated to ensure success.

Utilizing this methodology, CSFs in the context of this paper define key areas of instruction and design essential for an online class to be successful from a student’s perspective. Instructors implicitly know and consider these areas when setting up an online class and designing and directing activities and tasks important to achieving learning goals. CSFs make explicit those things good instructors may intuitively know and (should) do to enhance student learning. When made explicit, CSFs not only confirm the knowledge of successful instructors, but tap their intuition to guide and direct the accomplishment of quality instruction for entire programs. In addition, CSFs are linked with goals and objectives, helping generate a small number of truly important matters an instructor should focus attention on to achieve different thresholds of online success.

After a comprehensive literature review, an instrument was created to measure students’ perceptions about the importance of techniques and indicators leading to quality online classes. Items were designed to capture the major factors in the literature. The instrument was pilot studied during academic year 2017–18 with a 397 student sample, facilitating an exploratory factor analysis leading to important preliminary findings (reference withheld for review). Based on the pilot, survey items were added and refined to include seven groups of quality teaching factors and two groups of items related to students’ overall acceptance of online classes as well as a variable on their future online class enrollment. Demographic information was gathered to determine their effects on students’ levels of acceptance of online classes based on age, year in program, major, distance from university, number of online classes taken, high school experience with online classes, and communication preferences.

### Sample

This paper draws evidence from a sample of students enrolled in educational programs at Jack H. Brown College of Business and Public Administration (JHBC), California State University San Bernardino (CSUSB). The JHBC offers a wide range of online courses for undergraduate and graduate programs. To ensure comparable learning outcomes, online classes and face-to-face classes of a certain subject are similar in size—undergraduate classes are generally capped at 60 and graduate classes at 30, and often taught by the same instructors. Students sometimes have the option to choose between both face-to-face and online modes of learning.

A Qualtrics survey link was sent out by 11 instructors to students who were unlikely to be cross-enrolled in classes during the 2018–19 academic year.^1^ Approximately 2500 students were contacted, with some instructors providing class time to complete the anonymous survey. All students, whether they had taken an online class or not, were encouraged to respond. Nine hundred eighty-seven students responded, representing a 40% response rate. Although drawn from a single business school, it is a broad sample representing students from several disciplines—management, accounting and finance, marketing, information decision sciences, and public administration, as well as both graduate and undergraduate programs of study.

The sample age of students is young, with 78% being under 30. The sample has almost no lower division students (i.e., freshman and sophomore), 73% upper division students (i.e., junior and senior) and 24% graduate students (master’s level). Only 17% reported having taken a hybrid or online class in high school. There was a wide range of exposure to university level online courses, with 47% reporting having taken 1 to 4 classes, and 21% reporting no online class experience. As a Hispanic-serving institution, 54% self-identified as Latino, 18% White, and 13% Asian and Pacific Islander. The five largest majors were accounting & finance (25%), management (21%), master of public administration (16%), marketing (12%), and information decision sciences (10%). Seventy-four percent work full- or part-time. See Table 1 for demographic data.

**Table 1:** Demographic Information of the Participants (*n* = 987)

	Freq.	Valid %^**a**^
Age		
< =22	406	42%
23–29	348	36%
29–34	105	11%
35–40	47	5%
> =41	72	7%
Year in Program		
Freshman	8	1%
Sophomore	11	1%
Junior	359	36%
Senior	363	37%
Other	5	1%
Graduate	241	24%
Had HD/OL classes in high school		
Yes	167	17%
No	811	83%
Number of HD/OL classes taken		
0	215	21%
1–2	272	27%
3–4	224	22%
5–6	158	15%
7 and above	154	15%
Race		
Latino	525	54%
White	175	18%
African American	60	6%
Asian Pacific Islander	128	13%
Other	90	9%
Major		
Accounting & Finance	242	25%
Management	207	21%
Marketing	118	12%
Public Administration	73	7%
Information Decision Sciences	96	10%
Other (non-business students)	11	1%
MBA	75	8%
MPA	160	16%
MSA	2	0%
Working Status		
Not working	260	27%
Part-time	357	37%
Full-time	361	37%

^a^Percent eliminating missing values

### Measures and procedure

To increase the reliability of evaluation scores, composite evaluation variables are formed after an exploratory factor analysis of individual evaluation items. A principle component method with Quartimin (oblique) rotation was applied to explore the factor construct of student perceptions of online teaching CSFs. The item correlations for student perceptions of importance coefficients greater than .30 were included, a commonly acceptable ratio in factor analysis. A simple least-squares regression analysis was applied to test the significance levels of factors on students’ impression of online classes.

## Results

### Exploratory factor constructs

Using a threshold loading of 0.3 for items, 37 items loaded on seven factors. All factors were logically consistent. The first factor, with eight items, was labeled Teaching Presence. Items included providing clear instructions, staying on task, clear deadlines, and customized feedback on strengths and weaknesses. Teaching Presence items all related to instructor involvement during the course as a director, monitor, and learning facilitator. The second factor, with seven items, aligned with Cognitive Presence. Items included stimulating curiosity, opportunities for reflection, helping students construct explanations posed in online courses, and the applicability of material. The third factor, with six items, aligned with Social Presence defined as providing student-to-student learning opportunities. Items included getting to know course participants for sense of belonging, forming impressions of other students, and interacting with others. The fourth factor, with six new items as well as two (“interaction with other students” and “a sense of community in the class”) shared with the third factor, was Instructional Support which related to the instructor’s roles in providing students a cohesive learning experience. They included providing sufficient rehearsal, structured feedback, techniques for communication, navigation guide, detailed syllabus, and coordinating student interaction and creating a sense of online community. This factor also included enthusiasm which students generally interpreted as a robustly designed course, rather than animation in a traditional lecture. The fifth factor was labeled Basic Online Modality and focused on the basic technological requirements for a functional online course. Three items included allowing students to make online submissions, use of online gradebooks, and online grading. A fourth item is the use of online quizzes, viewed by students as mechanical practice opportunities rather than small tests and a fifth is navigation, a key component of Online Modality. The sixth factor, loaded on four items, was labeled Online Social Comfort. Items here included comfort discussing ideas online, comfort disagreeing, developing a sense of collaboration via discussion, and considering online communication as an excellent medium for social interaction. The final factor was called Interactive Online Modality because it included items for “richer” communications or interactions, no matter whether one- or two-way. Items included videoconferencing, instructor-generated videos, and small group discussions. Taken together, these seven explained 67% of the variance which is considered in the acceptable range in social science research for a robust model (Hair, Black, Babin, & Anderson, 2014). See Table 2 for the full list.

**Table 2:** Critical Success Factor Loading^a^

Survey Items	Factor 1 Teaching Presence	Factor 2 Cognitive Presence	Factor 3 Social Presence	Factor 4 Instructional Support	Factor 5 Basic Online Modality	Factor 6 Online Social Comfort	Factor 7 Interactive Modality
Online instructor provides clear instructions on how to participate in course learning activities.	0.8165						
Online instructor helps keep the course participants on task in a way that helped me to learn.	0.7801						
Online instructor clearly communicates important due dates/time frames for learning activities.	0.7651						
Online instructor provides feedback that helped me understand my strengths and weaknesses relative to the course’s goals and objectives.	0.7611						
Online instructor provides feedback in a timely fashion.	0.7293						
Online instructor clearly communicates important course goals.	0.6956						
Online instructor helps to focus discussion on relevant issues in a way that helped me to learn.	0.6379						
Online instructor encourages course participants to explore new concepts in this course.	0.6345						
Online course provide opportunities for meaningful reflection on course content		0.8525					
Online learning activities help me construct explanations/solutions in online courses.		0.8408					
Course activities stimulate my curiosity in online courses.		0.7206					
I can apply the knowledge created in online courses to my work or other non-class related activities.		0.6945					
I can utilize a variety of information sources to explore problems posed in online courses.		0.6628					
Online discussions are valuable in helping me appreciate different perspectives.		0.5518					
Posing problems in online courses increases my interest in course issues.		0.4220					
Getting to know other course participants gives me a sense of belonging in the course.			0.7551				
I am able to form distinct impressions of other course participants.			0.6484				
interaction with other students			0.6390	0.3125			
a sense of community in the class			0.5509	0.4327			
Online or web-based communication is an excellent medium for social interaction.			0.5026			0.3080	
including student goals			0.3950				
sufficient rehearsal of material, skills to be learned, etc.				0.7241			
instructor providing feedback				0.6852			
instructor having enthusiasm				0.6610			
the use of a variety of techniques to communicate and learn				0.4724			
navigation (e.g., being able to find what you want)				0.4010	0.3227		
syllabus (more detailed than in a face-to-face class)				0.3546			
allowing students to make online submissions					0.8136		
online gradebook					0.7464		
online grading of assignments by instructors					0.7409		
online quizzes					0.3308		
I felt comfortable participating in the course discussions.						0.8816	
I felt comfortable disagreeing with other classmates in online courses while still maintaining a sense of trust.						0.8149	
Online discussions help me to develop a sense of collaboration.						0.5301	
Zoom or other videoconference methods							0.9238
video lectures							0.7540
small groups discussions (chat rooms)							0.4920

^a^Seven factors explain 67% of the variance. Decimal places and loadings less than .30 omitted

To test for factor reliability, the Cronbach alpha of variables were calculated. All produced values greater than 0.7, the standard threshold used for reliability, except for system trust which was therefore dropped. To gauge students’ sense of factor importance, all items were means averaged. Factor means (lower means indicating higher importance to students), ranged from 1.5 to 2.6 on a 5-point scale. Basic Online Modality was most important, followed by Instructional Support and Teaching Presence. Students deemed Cognitive Presence, Social Online Comfort, and Online Interactive Modality less important. The least important for this sample was Social Presence. Table 3 arrays the critical success factor means, standard deviations, and Cronbach alpha.

**Table 3:** Priorities of CSFs and Factor Reliability

Rank^**a**^	Critical Success Factors	# of Items	n	Mean	Std Dev	Cronbach’s α
**1**	Basic Online Modality	5	818	1.4590	0.5521	0.7663
**2**	Instructional Support	8	816	1.6513	0.6403	0.8405
**3**	Teaching Presence	8	796	1.8270	0.7461	0.9233
**4**	Cognitive Presence	7	791	2.1715	0.7890	0.8957
**5**	Online Social Comfort	4	809	2.2464	0.9978	0.8602
**6**	Online Interactive Modality	3	818	2.2637	0.9892	0.7853
**7**	Social Presence	6	803	2.5571	0.9020	0.8611

^a^ Ranking is based on the average mean of students’ ranking importance of survey items: 1 = Very High, 2 = High, 3 = Neutral, 4 = Somewhat Low, and 5 = Very Low; Lower averages indicate greater importance

To determine whether particular subgroups of respondents viewed factors differently, a series of ANOVAs were conducted using factor means as dependent variables. Six demographic variables were used as independent variables: graduate vs. undergraduate, age, work status, ethnicity, discipline, and past online experience. To determine strength of association of the independent variables to each of the seven CSFs, eta squared was calculated for each ANOVA. Eta squared indicates the proportion of variance in the dependent variable explained by the independent variable. Eta squared values greater than .01, .06, and .14 are conventionally interpreted as small, medium, and large effect sizes, respectively (Green & Salkind, 2003). Table 4 summarizes the eta squared values for the ANOVA tests with Eta squared values less than .01 omitted.

**Table 4:** Eta Squared Values for Significant ANOVAs

Independent Variables	Critical Success Factors (from most to least important)						
	Basic online modality	Instruct-ional support	Teaching presence	Cognitive presence	Online Social Comfort	Online interactive modality	Social presence
*Graduate* vs. *Undergraduate* Graduates rate these factors higher than undergraduates		0.01	0.01	0.01		0.05	
*Age* Elder students rate this factor higher						0.03	
*Work Status* Working students rate these factors higher	0.02	0.02	0.02	0.03		0.01	0.01
*Ethnicity* Latino and White rate basic online modality and instructional support higher; API rate social presence higher	0.02	0.03					0.03
*Discipline* No significant difference found across 5 disciplines							
*Online Experience* Students who have taken more online classes rate all these factors higher	0.02	0.01	0.03	0.03	0.01	0.01	0.01

Eta Squared> 0.01 small effect, Eta Squared> 0.06 medium effect, Eta Squared> 0.14 large effect

While no significant differences in factor means among students in different disciplines in the College occur, all five other independent variables have some small effect on some or all CSFs. Graduate students tend to rate Online Interactive Modality, Instructional Support, Teaching Presence, and Cognitive Presence higher than undergraduates. Elder students value more Online Interactive Modality. Full-time working students rate all factors, except Social Online Comfort, slightly higher than part-timers and non-working students. Latino and White rate Basic Online Modality and Instructional Support higher; Asian and Pacific Islanders rate Social Presence higher. Students who have taken more online classes rate all factors higher.

In addition to factor scores, two variables are constructed to identify the resultant impressions labeled online experience. Both were logically consistent with a Cronbach’s α greater than 0.75. The first variable, with six items, labeled “online acceptance,” included items such as “I enjoy online learning,” “My overall impression of hybrid/online learning is very good,” and “the instructors of online/hybrid classes are generally responsive.” The second variable was labeled “face-to-face preference” and combines four items, including enjoying, learning, and communicating more in face-to-face classes, as well as perceiving greater fairness and equity. In addition to these two constructed variables, a one-item variable was also used subsequently in the regression analysis: “online enrollment.” That question asked: if hybrid/online classes are well taught and available, how much would online education make up your entire course selection going forward?

### Regression results

As noted above, two constructed variables and one item were used as dependent variables for purposes of regression analysis. They were online acceptance, F2F preference, and the selection of online classes. In addition to seven quality-of-teaching factors identified by factor analysis, control variables included level of education (graduate versus undergraduate), age, ethnicity, work status, distance to university, and number of online/hybrid classes taken in the past. See Table 5.

**Table 5:** Description of Variables

	Description
**Dependent Variable**	
Online Acceptance	Minus log sum of 1(strongly agree) to 5 (strongly disagree) rating of 6 items (Cronbach’s α = 0.8002): • I enjoy online learning. • My overall impression of hybrid/online learning is very good. • I often speak or communicate to others in online classes. • The instructors of online/hybrid classes are generally responsive. • Instructors reduce and catch cheating effectively in hybrid/online classes. • I am comfortable with online learning technologies.
F2F Preference	Minus log sum of 1(strongly agree) to 5 (strongly disagree) rating of 4 items (Cronbach’s α = 0.7525) • I enjoy face-to-face classes more. • I learn more in face-to-face classes. • I often speak or communicate to others in face-to-face classes. • I think that fairness and equity is better in face-to-face classes.
Selection Online Class	Log percent online class selection going forward
**Independent Variables**	
Undergraduate (vs. graduate)	Graduate (1) vs. Undergraduate (0)
Age	Log year of age
Race	White (1), African American (2), API (3), Latino (4), and Other (5)
Work Status	Full Time (2), Part Time (1), Not Work (0)
Distance to University	Log number of miles away from campus
Number of HD/OL Classes Taken	Log number of classes taken
Basic Online Modality	Minus Factor score
Instructional Support	Minus Factor score
Teaching Presence	Minus Factor score
Cognitive Presence	Minus Factor score
Online Social Comfort	Minus Factor score
Interactive Modality	Minus Factor score
Social Presence	Minus Factor score

When the ETA squared values for ANOVA significance were measured for control factors, only one was close to a medium effect. Graduate versus undergraduate status had a .05 effect (considered medium) related to Online Interactive Modality, meaning graduate students were more sensitive to interactive modality than undergraduates. Multiple regression analysis of critical success factors and online impressions were conducted to compare under what conditions factors were significant. The only consistently significant control factor was number of online classes taken. The more classes students had taken online, the more inclined they were to take future classes. Level of program, age, ethnicity, and working status do not significantly affect students’ choice or overall acceptance of online classes.

The least restrictive condition was online enrollment (Table 6). That is, students might not feel online courses were ideal, but because of convenience and scheduling might enroll in them if minimum threshold expectations were met. When considering online enrollment three factors were significant and positive (at the 0.1 level): Basic Online Modality, Cognitive Presence, and Online Social Comfort. These least-demanding students expected classes to have basic technological functionality, provide good opportunities for knowledge acquisition, and provide comfortable interaction in small groups. Students who demand good Instructional Support (e.g., rehearsal opportunities, standardized feedback, clear syllabus) are less likely to enroll.

**Table 6:** Summary of Multiple Regression Analysis: Online Class Enrollment

**Analysis of Variance**	**Online Class Enrollment**			
**Source**	**DF**	**Sum of Squares**	**Mean square**	**F Ratio**
Model	17	80.62	4.74	12.51
Error	686	260.15	0.38	**Prob > F**
C. Total	703	340.77		<.0001
**Parameter Estimates**				
**Term**	**Estimate**	**Std Error**	**t Ratio**	**Prob > |t|**
Intercept	3.66	0.08	44.19	<.0001***
Undergraduate (vs. graduate)	−0.02	0.03	−0.59	0.5546
Age	−0.04	0.06	−0.67	0.5000
African American (vs. White)	−0.08	0.08	−0.98	0.3297
Asian Pacific Islander (vs. White)	0.19	0.07	2.85	0.0045***
Latino (vs. White)	0.04	0.04	0.89	0.3752
Other Race (vs. White)	−0.12	0.07	−1.66	0.0978*
Nonworking (vs. full-time working)	0.06	0.04	1.58	0.1149
Nonworking (vs. part-time working)	−0.03	0.04	−0.69	0.4928
Distance to University	0.03	0.02	1.31	0.1922
Number of HD/OL Classes Taken	0.19	0.03	5.69	<.0001***
Basic Online Modality	0.08	0.03	2.45	0.0144**
Instructional Support	−0.10	0.03	−3.03	0.0026***
Teaching Presence	0.00	0.04	0.12	0.9023
Cognitive Presence	0.20	0.04	4.95	<.0001***
Online Social Comfort	0.06	0.03	1.65	0.0997*
Interactive Modality	0.00	0.03	−0.05	0.9591
Social Presence	0.04	0.03	1.24	0.2136

*p < .10, **p < .05, ***p < .01

Online acceptance was more restrictive (see Table 7). This variable captured the idea that students not only enrolled in online classes out of necessity, but with an appreciation of the positive attributes of online instruction, which balanced the negative aspects. When this standard was applied, students expected not only Basic Online Modality, Cognitive Presence, and Online Social Comfort, but expected their instructors to be highly engaged virtually as the course progressed (Teaching Presence), and to create strong student-to-student dynamics (Social Presence). Students who rated Instructional Support higher are less accepting of online classes.

**Table 7:** Summary of Multiple Regression Analysis: Online Class Acceptance

**Analysis of Variance**	**Online Acceptance**			
**Source**	**DF**	**Sum of Squares**	**Mean Square**	**F Ratio**
Model	17	37.19	2.19	37.97
Error	693	39.93	0.06	**Prob > F**
C. Total	710	77.13		<.0001
**Parameter Estimates**				
**Term**	**Estimate**	**Std Error**	**t Ratio**	**Prob > |t|**
Intercept	−2.61	0.03	−81.27	<.0001***
Undergraduate (vs. graduate)	0.00	0.01	−0.17	0.8672
Age	−0.01	0.02	− 0.50	0.6194
African American (vs. White)	−0.03	0.03	−1.07	0.2847
Asian Pacific Islander (vs. White)	0.03	0.03	0.98	0.3260
Latino (vs. White)	0.02	0.02	1.17	0.2414
Other Race (vs. White)	−0.02	0.03	−0.84	0.3992
Nonworking (vs. full-time working)	0.00	0.01	−0.06	0.9516
Nonworking (vs. part-time working)	0.00	0.02	−0.16	0.8714
Distance to University	0.00	0.01	−0.60	0.5516
Number of HD/OL Classes Taken	0.06	0.01	4.48	<.0001***
Basic Online Modality	0.05	0.01	4.05	<.0001***
Instructional Support	−0.05	0.01	−3.96	<.0001***
Teaching Presence	0.07	0.01	5.15	<.0001***
Cognitive Presence	0.11	0.02	6.77	<.0001***
Online Social Comfort	0.05	0.01	3.80	0.0002***
Interactive Modality	0.01	0.01	0.53	0.5972
Social Presence	0.06	0.01	4.61	<.0001***

***p < .01

Another restrictive condition was catering to the needs of students who preferred face-to-face classes (see Table 8). That is, they preferred face-to-face classes even when online classes were well taught. Unlike students more accepting of, or more likely to enroll in, online classes, this group rates Instructional Support as critical to enrolling, rather than a negative factor when absent. Again different from the other two groups, these students demand appropriate interactive mechanisms (Online Interactive Modality) to enable richer communication (e.g., videoconferencing). Student-to-student collaboration (Social Presence) was also significant. This group also rated Cognitive Presence and Online Social Comfort as significant, but only in their absence. That is, these students were most attached to direct interaction with the instructor and other students rather than specific teaching methods. Interestingly, Basic Online Modality and Teaching Presence were not significant. Our interpretation here is this student group, most critical of online classes for its loss of physical interaction, are beyond being concerned with mechanical technical interaction and demand higher levels of interactivity and instructional sophistication.

**Table 8:** Summary of Multiple Regression Analysis: F2F Preference

**Analysis of Variance**	**F2F Preference**			
**Source**	**DF**	**Sum of Squares**	**Mean Square**	**F Ratio**
Model	17.00	19.35	1.1384	9.36
Error	693.00	84.25	0.12158	**Prob > F**
C. Total	710.00	103.60		<.0001
**Parameter Estimates**				
**Term**	**Estimate**	**Std Error**	**t Ratio**	**Prob > |t|**
Intercept	−2.10	0.05	−44.99	<.0001***
Undergraduate (vs. graduate)	0.03	0.02	1.36	0.1729
Age	0.02	0.03	0.77	0.4417
African American (vs. White)	−0.01	0.05	−0.19	0.8460
Asian Pacific Islander (vs. White)	0.01	0.04	0.23	0.8174
Latino (vs. White)	−0.04	0.02	−1.52	0.1300
Other Race (vs. White)	0.04	0.04	0.89	0.3733
Nonworking (vs. full-time working)	0.03	0.02	1.58	0.1147
Nonworking (vs. part-time working)	−0.03	0.02	−1.49	0.1355
Distance to University	0.00	0.01	0.00	0.9997
Number of HD/OL Classes Taken	−0.11	0.02	−5.63	<.0001***
Basic Online Modality	0.01	0.02	0.74	0.4583
Instructional Support	0.07	0.02	3.83	0.0001***
Teaching Presence	−0.03	0.02	−1.35	0.1759
Cognitive Presence	−0.06	0.02	−2.68	0.0076***
Online Social Comfort	−0.06	0.02	−3.28	0.0011***
Interactive Modality	0.03	0.02	1.68	0.0937*
Social Presence	0.08	0.02	4.10	<.0001***

*p < .10, **p < .05, ***p < .01

## Discussion and study limitations

Some past studies have used robust empirical methods to identify a single factor or a small number of factors related to quality from a student’s perspective, but have not sought to be relatively comprehensive. Others have used a longer series of itemized factors, but have less used less robust methods, and have not tied those factors back to the literature. This study has used the literature to develop a relatively comprehensive list of items focused on quality teaching in a single rigorous protocol. That is, while a Beta test had identified five coherent factors, substantial changes to the current survey that sharpened the focus on quality factors rather than antecedent factors, as well as better articulating the array of factors often lumped under the mantle of “teaching presence.” In addition, it has also examined them based on threshold expectations: from minimal, such as when flexibility is the driving consideration, to modest, such as when students want a “good” online class, to high, when students demand an interactive virtual experience equivalent to face-to-face.

Exploratory factor analysis identified seven factors that were reliable, coherent, and significant under different conditions. When considering students’ overall sense of importance, they are, in order: Basic Online Modality, Instructional Support, Teaching Presence, Cognitive Presence, Social Online Comfort, Interactive Online Modality, and Social Presence. Students are most concerned with the basics of a course first, that is the technological and instructor competence. Next they want engagement and virtual comfort. Social Presence, while valued, is the least critical from this overall perspective.

The factor analysis is quite consistent with the range of factors identified in the literature, pointing to the fact that students can differentiate among different aspects of what have been clumped as larger concepts, such as teaching presence. Essentially, the instructor’s role in quality can be divided into her/his command of basic online functionality, good design, and good presence during the class. The instructor’s command of basic functionality is paramount. Because so much of online classes must be built in advance of the class, quality of the class design is rated more highly than the instructor’s role in facilitating the class. Taken as a whole, the instructor’s role in traditional teaching elements is primary, as we would expect it to be. Cognitive presence, especially as pertinence of the instructional material and its applicability to student interests, has always been found significant when studied, and was highly rated as well in a single factor. Finally, the degree to which students feel comfortable with the online environment and enjoy the learner-learner aspect has been less supported in empirical studies, was found significant here, but rated the lowest among the factors of quality to students.

Regression analysis paints a more nuanced picture, depending on student focus. It also helps explain some of the heterogeneity of previous studies, depending on what the dependent variables were. If convenience and scheduling are critical and students are less demanding, minimum requirements are Basic Online Modality, Cognitive Presence, and Online Social Comfort. That is, students’ expect an instructor who knows how to use an online platform, delivers useful information, and who provides a comfortable learning environment. However, they do not expect to get poor design. They do not expect much in terms of the quality teaching presence, learner-to-learner interaction, or interactive teaching.

When students are signing up for critical classes, or they have both F2F and online options, they have a higher standard. That is, they not only expect the factors for decisions about enrolling in noncritical classes, but they also expect good Teaching and Social Presence. Students who simply need a class may be willing to teach themselves a bit more, but students who want a good class expect a highly present instructor in terms responsiveness and immediacy. “Good” classes must not only create a comfortable atmosphere, but in social science classes at least, must provide strong learner-to-learner interactions as well. At the time of the research, most students believe that you can have a good class *without* high interactivity via pre-recorded video and videoconference. That may, or may not, change over time as technology thresholds of various video media become easier to use, more reliable, and more commonplace.

The most demanding students are those who prefer F2F classes because of learning style preferences, poor past experiences, or both. Such students (seem to) assume that a worthwhile online class has basic functionality and that the instructor provides a strong presence. They are also critical of the absence of Cognitive Presence and Online Social Comfort. They want strong Instructional Support and Social Presence. But in addition, and uniquely, they expect Online Interactive Modality which provides the greatest verisimilitude to the traditional classroom as possible. More than the other two groups, these students crave human interaction in the learning process, both with the instructor and other students.

These findings shed light on the possible ramifications of the COVID-19 aftermath. Many universities around the world jumped from relatively low levels of online instruction in the beginning of spring 2020 to nearly 100% by mandate by the end of the spring term. The question becomes, what will happen after the mandate is removed? Will demand resume pre-crisis levels, will it increase modestly, or will it skyrocket? Time will be the best judge, but the findings here would suggest that the ability/interest of instructors and institutions to “rise to the occasion” with quality teaching will have as much effect on demand as students becoming more acclimated to online learning. If in the rush to get classes online many students experience shoddy basic functional competence, poor instructional design, sporadic teaching presence, and poorly implemented cognitive and social aspects, they may be quite willing to return to the traditional classroom. If faculty and institutions supporting them are able to increase the quality of classes despite time pressures, then most students may be interested in more hybrid and fully online classes. If instructors are able to introduce high quality interactive teaching, nearly the entire student population will be interested in more online classes. Of course students will have a variety of experiences, but this analysis suggests that those instructors, departments, and institutions that put greater effort into the temporary adjustment (and who resist less), will be substantially more likely to have increases in demand beyond what the modest national trajectory has been for the last decade or so.

There are several study limitations. First, the study does not include a sample of non-respondents. Non-responders may have a somewhat different profile. Second, the study draws from a single college and university. The profile derived here may vary significantly by type of student. Third, some survey statements may have led respondents to rate quality based upon experience rather than assess the general importance of online course elements. “I felt comfortable participating in the course discussions,” could be revised to “comfort in participating in course discussions.” The authors weighed differences among subgroups (e.g., among majors) as small and statistically insignificant. However, it is possible differences between biology and marketing students would be significant, leading factors to be differently ordered. Emphasis and ordering might vary at a community college versus research-oriented university (Gonzalez, 2009).

## Data Availability

We will make the data available.
